# Dissection of Kinesin's Processivity

**DOI:** 10.1371/journal.pone.0004612

**Published:** 2009-02-26

**Authors:** Sarah Adio, Johann Jaud, Bettina Ebbing, Matthias Rief, Günther Woehlke

**Affiliations:** 1 Physics Department E22, Technical University Munich, Garching, Germany; 2 Munich Center for Integrated Protein Sciences, Munich, Germany; Griffith University, Australia

## Abstract

The protein family of kinesins contains processive motor proteins that move stepwise along microtubules. This mechanism requires the precise coupling of the catalytic steps in the two heads, and their precise mechanical coordination. Here we show that these functionalities can be uncoupled in chimera of processive and non-processive kinesins. A chimera with the motor domain of Kinesin-1 and the dimerization domain of a non-processive Kinesin-3 motor behaves qualitatively as conventional kinesin and moves processively in TIRF and bead motility assays, suggesting that spatial proximity of two Kinein-1 motor domains is sufficient for processive behavior. In the reverse chimera, the non-processive motor domains are unable to step along microtubules, despite the presence of the Kinesin-1 neck coiled coil. Still, ATP-binding to one head of these chimera induces ADP-release from the partner head, a characteristic feature of alternating site catalysis. These results show that processive movement of kinesin dimers requires elements in the motor head that respond to ADP-release and induce stepping, in addition to a proper spacing of the motor heads via the neck coiled coil.

## Introduction

Kinesins have been found as motor proteins that move along microtubules at the expense of ATP [1]. The founding member belongs to the class of Kinesin-1 (formerly conventional kinesin) and moves processively along microtubules, meaning that it moves stepwise over long distances without detachment from the filament. Its movement is based on the coordinated action of two motor heads that bind one after another to the microtubule, a mechanism termed hand-over-hand motility [2]. This mechanism requires precisely coordinated microtubule affinities of the two motor domains. Otherwise, the motor would dissociate from the microtubule in between steps, or stick to the filaments in a state with both motor domains in a strong microtubule binding state [2]–[6].

This mechanism implies that conventional kinesin motors have to be dimeric in order to move processively [7]. However, although dimerization of kinesin via a two-stranded coiled coil structure is a necessary prerequisite for hand-over-hand motility, it is not sufficient. This has become clear by studies on Ncd, a Kinesin-14 motor, and NcKin3, a fungal Kinesin-3 motor [8]–[16]. These motors are dimers but not processive.

Ncd has been studied extensively, in particular with respect to its direction of motion. In contrast to most kinesins, Ncd and other Kinesin-14 motors move to microtubule minus ends. The elements responsible for Ncd's direction of motion have been mapped by several groups, who have shown that the linkage between motor domain and coiled coil plays a central role [17]–[19]. These studies used chimeric motors with parts of Ncd and parts of Kinesin-1, and, together with crystallographic studies, solved the fundamentals of directionality [11], [13]. However, like directionality and velocity, processivity is a fundamental quality of motor proteins. The structural prerequisites for this property have not been mapped yet, in part because processivity assays are technically challenging, and the only rigorously characterized non-processive motor (Ncd) moves in the opposite direction as Kinesin-1. This makes any analysis more complicated, if not impossible. Accordingly, to our knowledge no previous study has addressed the question whether Kinesin-1/Ncd chimera move processively.

Therefore, the discovery and characterization of the non-processive kinesin plus-end motor NcKin3 for the first time made it possible to compare a processive and a non-processive kinesin motors with the goal of elucidating prerequisites for processivity and hand-over-hand motility. NcKin3 binds only with one head to the microtubule and performs a single round of ATP hydrolysis before it detaches [16]. The second head hydrolyses the bound nucleotide at the basal rate in a microtubule independent manner, and plays a kinetically passive role for motility. This result came as a surprise as the domain organization is quite similar to Kinesin-1, in particular with respect to the location of the coiled coil dimerization domain. It posed the question why one type of motors (Kinesin-1) moves in a stepwise fashion, the other one (NcKin3) does not, and which parts of the molecules provide the functionality of stepping and coupled kinetic cycles. To locate these parts, we generated chimeric motors containing elements of processive NcKin1 and non-processive NcKin3. We analyzed the motile properties of these novel motors using single molecule fluorescence and single bead optical trapping assays, as well as transient kinetic techniques.

## Results

### Processive motility of chimera Head1/Neck3 in TIRF assays

**Figure 1:**
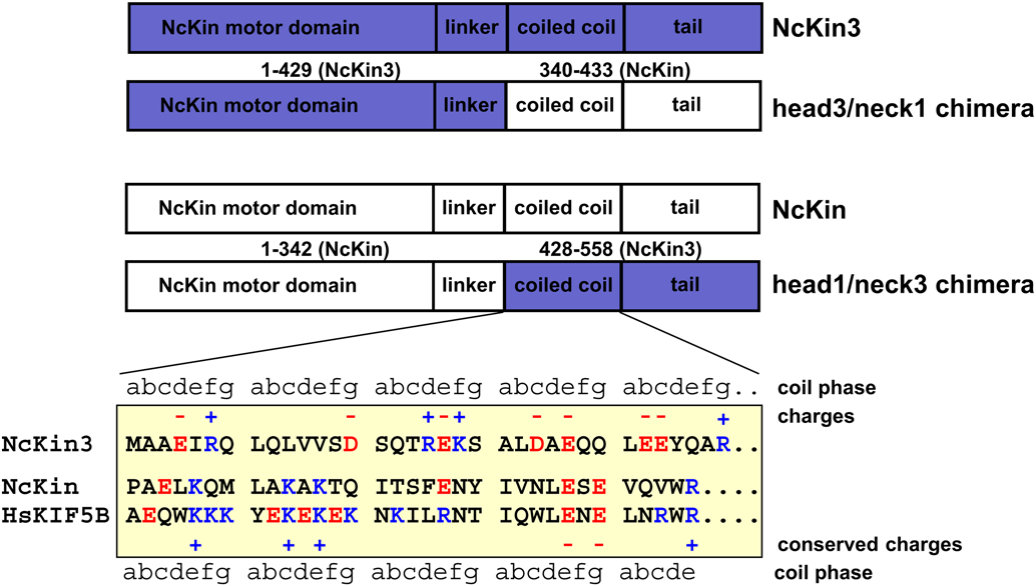
Design of chimeric kinesin constructs. The upper part of the figure shows the domain organization and the fusion points of chimeric kinesin motors. Below, the neck regions are shown in detail. The coiled coil assignment for the kinesin-1 members NcKin and HsKIF5B (ubiquitous conventional kinesin) is taken from the crystal structure 3KIN, the NcKin3 coiled coil is predicted by computer algorithms and experimental data [16]. Positively charged residues are blue, negatively charged red.

To map elements of kinesin involved in processive hand-over-hand motility, we used chimera of the processive, fungal NcKin Kinesin-1, and the non-processive NcKin3 Kinesin-3 motor from *Neurospora crassa* (Figure 1). NcKin is a ‘conventional’, processive Kinesin-1 and uses a mechanism as other Kinesin-1 motors, NcKin3 a non-processive Kinesin-3 that presumably uses a power stroke mechanism to generate microtubule plus-end directed movement [16], [20]–[22]. One construct contained NcKin's motor head in front of the neck of its the non-processive counterpart (Head1/Neck3), the other one contained the motor head of the non-processive motor in front of the neck and partial stalk of NcKin3 (Head3/Neck1). The fusion points were chosen such that motor heads contained the catalytic motor domains and their native neck-linkers (see [23] for a definition of domain borders).

**Figure 2:**
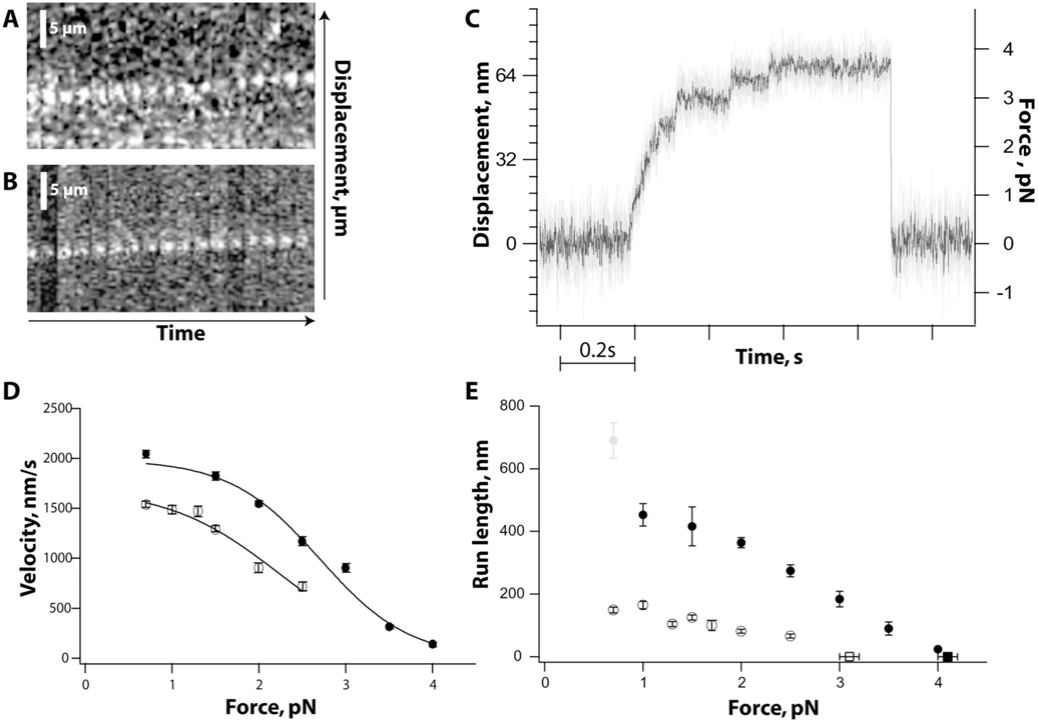
Single-molecule properties of the Head1/Neck3 chimera. (A) Kymographs show displacements of a single fluorophore-labeled Head1/Neck3 motor along the microtubule. (B) Wildtype NcKin was used as a control. A single motor moves continuously on a microtubule with a velocity essentially identical to the average velocity under multiple motor conditions. Motors were observed for 5 sec with an integration time of 200 ms at an ATP concentration of 20 µM. (C) Record of a microscopic latex bead captured by an optical trap and attached to a single Head1/Neck3 protein. Upon binding to surface immobilized microtubules the motor moves stepwise along the filament until it detaches at a stall force of 3.1 pN. Near stall resolution of 8 nm steps indicate hand-over-hand motility. (D) Head1/Neck3 velocities under load. Average velocities v (mean±s.e.m.) of NcKin wild type motor (closed circles [22] and additional data points) and Head1/Neck3 chimera (open circles) are plotted versus applied load. Measurements were performed under constant force using a force feedback-controlled optical trap. The average velocities of both motors drop with increasing external force. Data were fitted (solid line) by Bell's equation [47] assuming a kinetic model with one force-independent rate-limiting transition, and one force-dependent rate. The transition state position of approximately 8 nm suggests that the step is dominated by diffusive search. (E) Head1/Neck3 runlength under load. Average runlengths (mean±SE) of NcKin wild type motor (closed circles, data from [22], model according to [47]) chimera (open circles) are plotted versus applied load. At all forces tested the chimera has a reduced runlength compared to wild type NcKin. Imperfect head-head coordination is most likely the reason for a higher detachment probability and the decrease in processivity.

To identify the functional consequences the domain swaps, we tested the mutants along with wildtype reference constructs in motility assays. Fluorescently labeled kinesin motors were observed over time in a TIRF-microscope to detect processive kinesin runs. As the NcKin wildtype reference, the Head1/Neck3 mutant turned out to move processively (Figure 2A and 2B). Analyses of this assay showed that this chimera moved at a velocity of 1.40±0.03 µm/s (mean±s.e.m.) over up to 3 µm. The average runlength of the chimera was 0.67±0.13 µm and thus at least 2–3-fold shorter than for wildtype NcKin (∼1.8 µm, [21]). We show below that these runs were actually caused by stepwise moving motor heads.

The velocities observed in TIRF assays were comparable to those in multi-motor gliding assays where the motor was attached to the coverslip surface, and microtubules slid over the surface (Table 1). In contrast to the NcKin3 reference, the microtubule gliding velocity was basically insensitive to the coating density (1.48±0.01 µm/s at 1.6 µM Head1/Neck3 concentration, 1.61±0.03 µm/s at 0.3 µM Head1/Neck3 concentration), hinting at mechanistic differences between wildtype NcKin3 and the Head1/Neck3 chimera.

**Table 1:** Steady-state ATPase parameters and multiple motor gliding velocities of chimeric and wild type motors.

	Steady state ATPase			Motility		
	k_cat_, s^−1^	K_0.5,Mt_, µM	K_M,ATP_, µM	V_gld_, µm/s (multi-motor assay)	V_gld_, µm/s (TIRF assay)	Runlength, µm (TIRF assay)
**NcKin3 ** [**16**]	23.2±8.0	1.0±1.2	4.0±0.9	0.52±0.04	n/a	n/a
**Head3/Neck1**	23.2±1.0	0.2±0.12	49.1±1.3	0.58±0.04	n/a	n/a
	n = 4	n = 2	n02	n = 90		
**NcKin**	66.9±18.1	2.2±1.4	64.5±20.4	2.29±0.01	2.38±0.01	1.75±0.09
	n = 4	n = 2	n = 2	n = 60	n = 16	[48]
**Head1/Neck3**	99.6±15.5	2.1±0.4	33.1±4.6	1.61±0.03	1.40±0.03 µm/s	0.67±0.13 µm
	n = 7	n = 3	n = 4	n = 120	n = 80	n = 74

The ATPase activity of the Head1/Neck3 chimera showed a turnover of k_cat_ = 99±16 s^−1^ of the microtubule-activated enzyme, with a half-maximal activation constant for microtubules of K_0.5,Mt_ = 2.1±0.4 µM. Assuming a construct moving hand-over-hand with two coupled heads, the gliding velocity calculated from k_cat_ and step size is 99 s^−1^ · 8.2 nm∼0.8 µm/s. This is two-fold slower than the measured gliding velocity. The reasons why the chimera moves faster than expected from kinetic data are unclear. Wildtype NcKin1 shows an even more pronounced discrepancy (typically k_cat_∼60 s^−1^ and v_gld_∼2 µm/s), which has been attributed to regulatory effects [24], [25].

The ATPase and motility data thus show that the motor domain of NcKin performs its native activity (i.e.: processive motility) even in the presence of the non-native neck domain, and is not dominated by the neck of NcKin3. These data also show that the specific neck of NcKin3 adopts a structure capable of joining two kinesin motor domains in a way that promotes processive motility.

### Quantitative differences between Kinesin-1 and chimera Head1/Neck3

To test whether the processive motility of the Head1/Neck3 chimera was due to a stepwise progression mechanism, we performed bead assays in a laser trap microscope. This assay can follow the position of the motor to nm-precision and thus is capable of detecting 8-nm steps [26], [27]. In fact, the Head1/Neck3 chimera behaved qualitatively similar to conventional NcKin and showed processive runs caused by single kinesin motors up to stall forces of more than 3 pN (Figure 2C). Quantitatively, however, Head1/Neck3 behaved differently from the wildtype reference construct. The force-velocity relationship of this mutant in a force clamp showed slower velocities of the mutant under each load (Figure 2D). The wildtype reference curve ([22] extended by new data) could be fit with a model including two rate-limiting steps, one force-dependent and one force-independent [28]. The load-independent step occurred at a rate of 249±3 s^−1^, corresponding to a gliding velocity of ∼2 µm/s if the motor performs steps of 8 nm. The force-dependent step occurred at a rate of 46,600±9,800 s^−1^ and was associated with a transition state position of 7.7±0.3 nm. The force-velocity dependence of the chimera could be fit by the same model, suggesting an identical motor mechanism. Here, the load-independent rate (212±8 s^−1^) and the transition state position of the load-dependent rate were similar (6.3±0.7 nm), but the rate of the load-dependent step was much slower (7,000±3,000 s^−1^).

The rates in this model most likely reflect the ADP release rate (slow, force-independent component) and a composite rate (fast, force-dependent) that is dominated by the stepping process. The force-independent rate closely resembles the k_cat_ of the NcKin motor domain [29], and previous studies support the identification of this rate with ADP release [30]. The force-dependent rate is most likely a compound rate resulting from a number of fast rates within the reaction cycle. As in myosin V, this rate may be dominated by the diffusive search for the next microtubule-binding site [31]. This could be the explanation for the almost sevenfold decrease in the force-dependent rate in the Head1/Neck3 mutant. According to this model, the neck of the non-processive kinesin hinders the diffusive search for the next binding site.

As a consequence, the motor dwells longer in a one-head-bound state that is more sensitive to detachment. In fact, the runlength of the chimera was affected stronger by backward loads (Figure 2E). At all forces, the runlengths were roughly fivefold shorter than wildtype, suggesting a general steric defect that impedes stepping rather than a compliance defect as observed in mutants with a stiffer neck coiled-coil [22]. Still, the force-dependent velocity and runlength behavior is consistent with a hand-over-hand mechanism, and supports previous models that make the neck responsible for modulations of processive motility properties [22], [32], [33].

### Behavior of the Head3/Neck1 chimera

After the demonstration that the Head1/Neck3 chimera is a processive motor, we investigated the properties of the converse construct, Head3/Neck1. In microscopic motility assays, this construct never showed any typical indications of processive motility: characteristic pivoting at low motor densities was not observed, microtubules moved farther than their length, and in contrast to the NcKin3 reference construct the chimera was not accelerated by higher motor coating densities. In fact, the gliding velocity decreased slightly from 0.58±0.04 µm/s at low densities to 0.45±0.01 µm/s at high densities, indicating mutual hindrance of motors among each other. Overall, the maximal gliding velocity of the Head3/Neck1 chimera was very similar to the NcKin3 wildtype and almost threefold slower than that of the Head1/Neck3 chimera, suggesting largely different mechanisms between the mutant motors.

**Figure 3:**
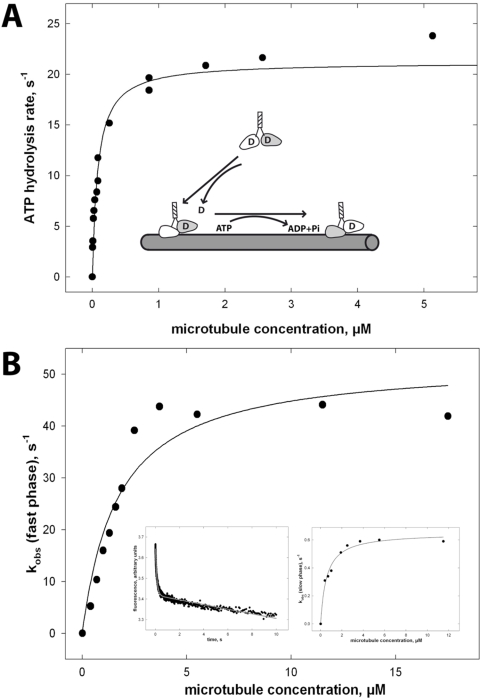
k_bi_ ratio of non-processive Head3/Neck1. (A) The steady state ATPase rate of the Head3/Neck1 motor was measured at variable microtubule concentrations. Based on the concentrations of polypeptide chains the k_cat_ was 23.2 s^−1^ and the microtubule concentration for half maximal activation (K_0.5,MT_) was 0.2 µM, the apparent binding rate between Head3/Neck1 and microtubules (k_bi_(ATP) = k_cat_/K_0.5,MT_) was 116.0 µM^−1^·s^−1^ (Table 1). (B) To determine the ADP release rate mant-ADP loaded Head3/Neck1 was mixed with microtubules in a stopped-flow apparatus. The left inset shows an example of the fluorescence decay at 1.2 µM microtubules. The reaction was fitted with a double-exponential function (grey line). Each data point is an average of at least five individual stopped-flow traces. Rates were plotted against the microtubule concentration. The hyperbolic fit of the data revealed a maximal ADP release rate of k_max_ = 52.5±4.5 s^−1^ with a K_0.5, MT_ = 1.72±0.43 µM for the fast rate, and k_max_ = 0.65±0.02 s^−1^/K_0.5, MT_ = 0.53±0.08 µM for the slow phase (right inset). The comparison of the bimolecular rate of the fast rate in this assay (k_max_/K_0.5,MT_ = 30.5 µM^−1^ s^−1^) with the apparent rate in steady state reveals a low biochemical processivity index of 3–4 ATPs hydrolyzed per microtubule encounter by the Head3/Neck1 chimera.

To correlate the gliding velocity with ATP turnover, we determined the microtubule-activated steady state ATPase rate of Head3/Neck1. The chimera had a k_cat_ of 23.2±1.0 s^−1^ and a K_0.5,Mt_ of 0.2±0.1 µM (Figure 3A). Assuming a hand-over-hand mechanism with steps of 8 nm, this would lead to a gliding velocity of 0.19 µm/s, threefold lower than actually observed. Hence, at first sight the chimera resembles the wildtype NcKin3 motor in its gliding velocity and ATPase turnover (0.6 µm/s with k_cat_ = 23.2 s^−1^), suggesting a common mechanism. Closer inspection, however, shows that this is not the case.

### Differences between Head3/Neck1 and NcKin3 wildtype

**Figure 4:**
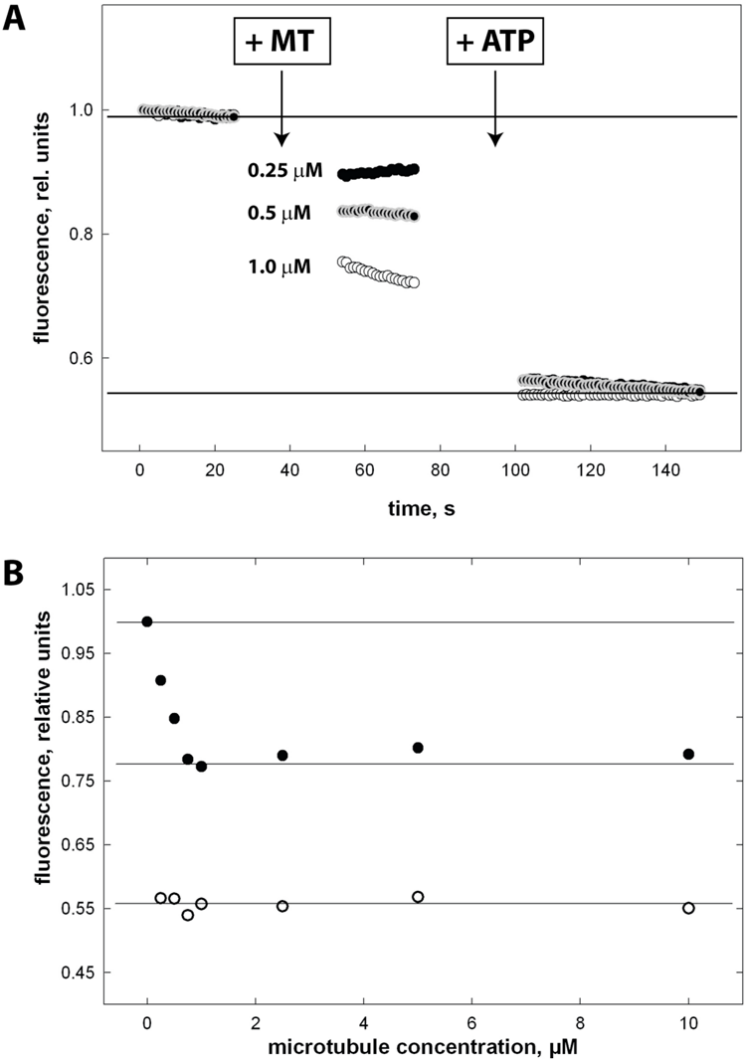
Microtubule activated ADP release from the Head3/Neck1 chimera. Panel A shows the normalized time courses of mantADP release from Head3/Neck1-mantADP complex after addition of various amount of microtubules (final concentration c = 0.25 µM closed circles, c = 0.5 µM grey circles and c = 1.0 µM open circles) and 1 mM ATP. Panel B summarizes the fluorescence amplitudes after addition of microtubules (closed circles) and ATP (open circles).

There are three major differences between the Head3/Neck1 chimera and NcKin3 wildtype. (i) First, one important difference is the biochemical processivity, determined by comparison of steady state ATP turnover and microtubule-induced ADP release [34]. From the steady state ATPase parameters it follows that k_cat_/K_0.5,Mt_ of the Head3/Neck1 chimera is 23.2/0.2 µM^−1^·s^−1^ = 116.0 µM^−1^·s^−1^ (Fig 3A and Table 1). The fast phase of the microtubule-induced mant-ADP release rate in stopped-flow assays showed k_max_/K_0.5,Mt_ = 52.5±s^−1^/1.7 µM = 30.9 µM^−1^·s^−1^ (Figure 3B). The slow phase is probably due to background drift. The ratio of these values suggests a biochemical processivity of k_bi,ratio_ = 116.0/30.9 = 3.8, indicating that Head3/Neck1 remains attached to the microtubule filament for 3–4 catalytic cycles. This is more than the NcKin3 reference that detaches after one ATP turnover [16].

(ii) Secondly, apart from quantitative deviations in the k_bi,ratio_ the Head3/Neck1 chimera showed fundamental differences to the NcKin3 reference construct. NcKin3 uses only one of its two motor heads during catalysis and retains the other head in an inactivated state [16]. The Head3/Neck1 chimera does not, as shown by mant-ADP release assays. In these assays, we bound the mant-ADP charged Head3/Neck1 chimera to microtubules and waited until equilibrium, which resulted in the release of one mant-ADP ligand from the bound head. Subsequent addition of ATP induced the release of the second mant-ADP ligand (Figure 4A). The existence of a stable single-head interacting intermediate of the chimera before ATP addition was confirmed by titration over a huge range of microtubule concentrations (Figure 4B). Starting from theses single-head attached kinesin microtubule complexes we measured the rate of ATP induced mant-ADP release from the second head in a stopped-flow experiment. The maximum rate at saturating ATP concentrations was k_max_ = 47±8 s^−1^ with a half-maximal activation constant K_m,ATP_ = 60±16 µM (Table 2). ATP-dependent ADP release from the single-head attached intermediate does not occur in the wildtype NcKin3 reference construct whose second passive head does not show ATP-dependent interaction with microtubules [16].

**Table 2:** Comparison of ADP release and detachment kinetics of wildtype NcKin3 and the NcKin-Tail chimera.

	1^st^ ADP release		2^nd^ ADP release		Detachment	
	k_max_, s^−1^	K_0.5,Mt_, µM	k_max_, s^−1^	K_0.5,Mt_, µM	k_max_, s^−1^	K_0.5,ATP_, µM
**NcKin3 ** [**16**]	7.1±2.0	0.94±0.04	0.02	------	22.19±7.64	2.22±2.75
**Head3/Neck1**	fast phase 52.5±4.5	1.72±0.43	47±8	60±16	0.47±0.03	105.0±26.6
			n = 2	n = 2	n = 2	n = 2
	slow phase 0.65±0.02	0.53±0.08				
	n = 2	n = 2				

(iii) The third significant difference between Head3/Neck1 and its non-processive reference was the microtubule detachment rate (Figure 5). Whereas NcKin3 showed ATP-dependent microtubule-detachment at a rate essentially identical to k_cat_ (23 s^−1^, [16]), the Head3/Neck1 chimera was two orders of magnitude slower (k_off_ = 0.47±0.03 s^−1^), suggesting that the chimera stays in a microtubule-associated state after each ATPase cycle.

**Figure 5:**
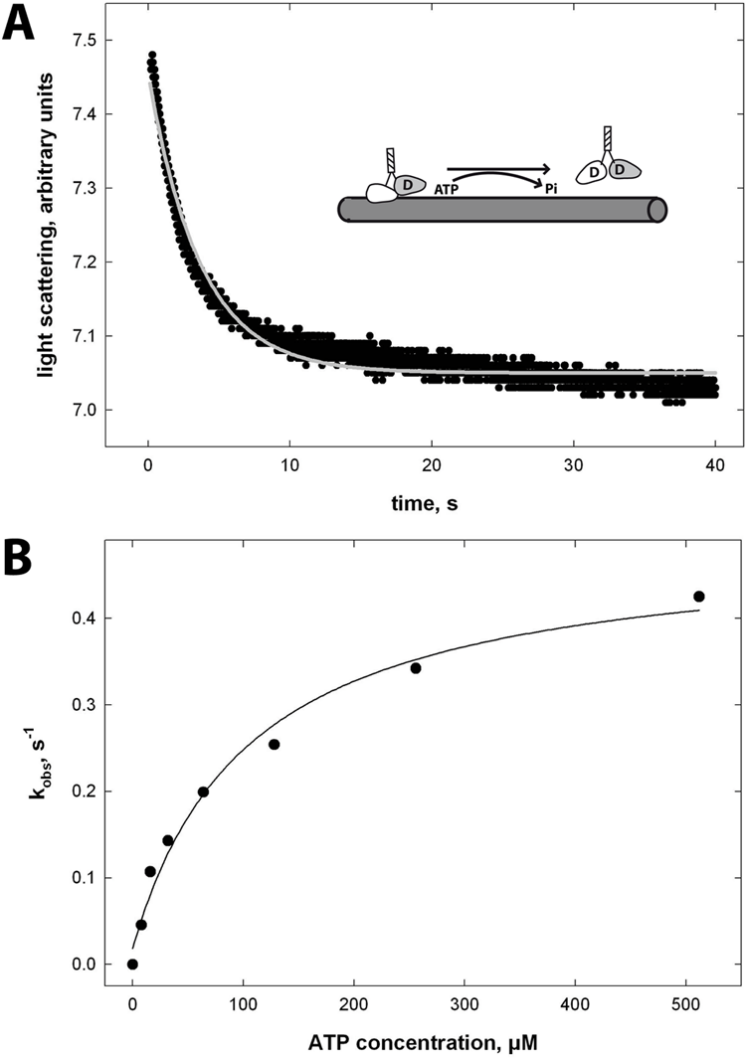
Dissociation of the Head3/Neck1 microtubule complex. The dissociation of the pre-formed Head3/Neck1 microtubule complex was induced by ATP in a stopped-flow apparatus (inset panel A) and followed by the change of the light scattering signal. (A) The graph shows a representative average from 5 traces. The grey curve is a mono-exponential fit to the data that was used to derive k_obs_. (B) Graph B shows the hyperbolic dependence of k_obs_ on the ATP concentration, with a k_max_ of 0.47 s^−1^ and a K_1/2_ of 105.0 µM ATP.

**Figure 6:**
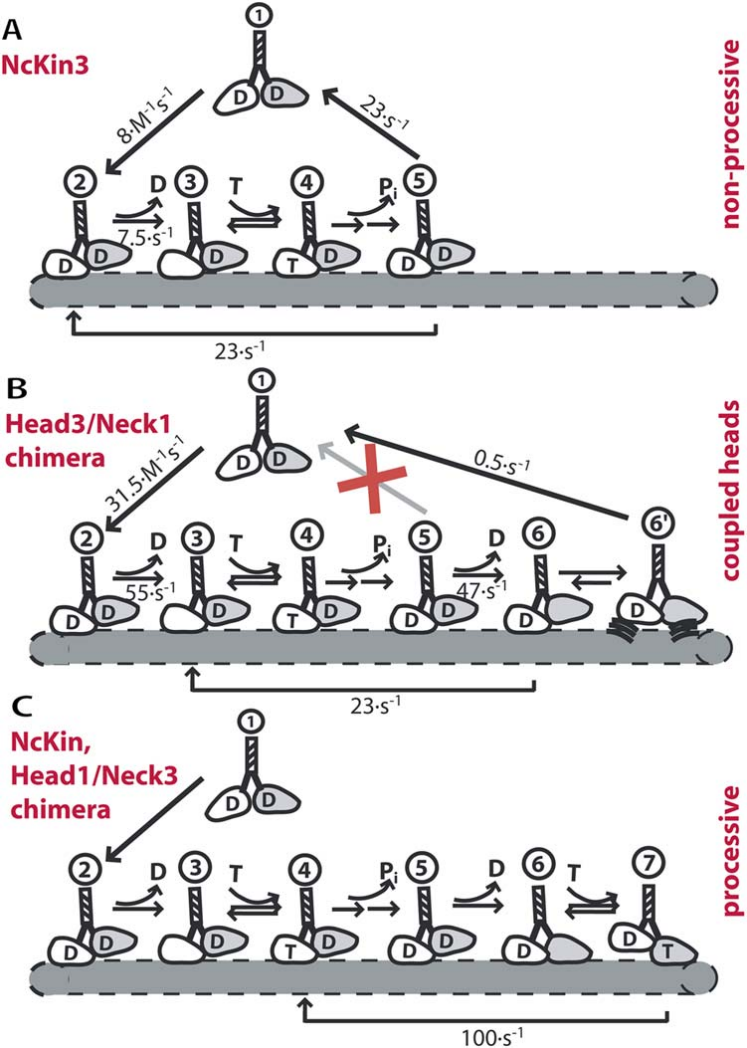
Kinetic models of constructs of this study. The figure summarizes the different mechanisms of processive and non-processive kinesins. (A) The non-processive Kinesin-3 reference construct NcKin3 binds to microtubules and detaches after one hydrolysis cycle. (B) The Head3/Neck1 chimera is unable to detach from the filament after one catalytic cycle (intermediate 5). At this state, the kinetic pathway branches and part of the enzymes cycle through ATP hydrolysis, part of them dwell in a long-lived microtubule-bound state (6′) before detachment. (C) NcKin and Head1/Neck3 mutant are processive enzymes that proceed after intermediate 5 via a double-head bound intermediate to the initial state where they are able to repeat the catalytic cycle.

These differences between the Head3/Neck1 mutant and wildtype NcKin3 indicate that the proper NcKin3 neck domain is necessary to inactivate one of the motor domains of wildtype NcKin3, and to achieve microtubule detachment of NcKin3 after each powerstroke. The long dwell time of the Head3/Neck1 chimera at the microtubule suggests a kinetic dead-end that shows weak microtubule affinity and is unable to step forward. Therefore, the chimera lacks tight coupling of ATPase activity and progression along the microtubule.

## Discussion

The most important result of our analysis is that kinesin motors contain two separable activities that together lead to processive, stepwise motility. The first activity is associated with the motor domain and causes processivity of the Head1/Neck3 chimera that moves stepwise and processively along microtubules. The chimera's properties demonstrate that the motor core of Kinesin-1 contains elements that couple the ATP turnover to repetitive stepping (similar conclusions have been drawn recently from a completely different experimental approach [35]). These elements are obviously absent in the reverse construct, Head3/Neck1 that still shows mant-ADP release upon ATP-binding to the microtubule-bound partner head, but is unable to move in a stepwise fashion. Given the identical length and the similarity of the neck-linkers of Kinesin-1 and NcKin3 (as used in the chimera), it is unlikely that the failure of stepping is simply due to a linkage between the motor domains that is too short to bridge 8 nm. The microtubule-detachment rate of this chimera that is ∼50-fold slower than k_cat_ and ∼100-fold slower than mant-ADP release indicates that the motor head that just released ADP is unable to inhibit the ATP-turnover of the microtubule-bound head, as would be the case in fully processive kinesins. Hence, the motor head (comprising catalytic core and neck-linker) contains elements ensuring coupling of catalysis and stepping.

Still, elements outside the motor domain are important, as they are able to couple nucleotide binding and release of the two heads: NcKin3 detaches from the microtubule after one ATPase cycle, and is therefore unable to step “hand-over-hand”. However, Kinesin-1's neck domain can restore the characteristic inter-head coupling of processive kinesins in NcKin3. This is shown by the quick, microtubule-dependent release of ADP from the “second” motor domain of the chimera Head3/Neck1 in stopped-flow experiments, not observed in wildtype NcKin3. Still, the chimera does not move processively. The chimera is thus a partially functional motor that shows a gain-of-function that reveals an important key prerequisite for alternating site catalysis and processivity.

Recently, the concept of ‘gating’ has become popular to explain kinesin's mechanism. It has been proposed to look at kinesin's kinetic cycle in terms of gates that have to be passed to reach the next kinetic intermediate [5], [6]. One of these gates leads from the pre-step to the post-step configuration, and this gate may depend on the presence or absence of elements identified in this study. Figure 5 illustrates the critical transitions in the catalytic cycles of the constructs investigated in our study. Especially important for processivity is the control point of the intermediate that contains two microtubule-bound heads (Figure 6, intermediate 6). One model assumes that the leading motor head of conventional kinesin (but also of myosin V and VI) is inhibited in its ATP binding as long as the lagging head remains filament-bound [5], [36], [37]. That way, the motor prevents premature detachment and achieves directed stepping to the microtubule plus-end (intermediate 6→7 in Figure 6). Our data show that - like the processive NcKin reference - the Head1/Neck3 chimera possesses this particular control point, demonstrating that the control point has its structural basis in the motor domain.

Another essential control point for processivity is the transition between intermediates 5 and 6. The structural element responsible for this gate, however, is neither located in the motor core nor in a specific neck feature, as seen by the Head3/Neck1 chimera. Apparently, any stable connection that allows proper spacing and steric coupling (‘communication’) of the motor heads leads to processive motility (cf. [22], [32]). Stopped-flow experiments clearly showed that the rigor complex (intermediate 3) releases mant-ADP upon ATP binding. This control point is thus necessary but not sufficient for processive motility.

The Head3/Neck1 chimera also demonstrates that the specific NcKin3 neck domain is responsible for detachment of the non-processive NcKin3 motor (transition 5 to 1 in Figure 6A). This chimera also confirms our previous hypothesis that the neck domain of NcKin3 inactivates the second head [16]. Both of these properties are absent in the chimera, although the NcKin3 neck, as well as the NcKin neck, forms a two-stranded α-helical coiled coil. Apparently, residues specific for NcKin3 control the detachment kinetics of the wildtype motor. The absence of the native NcKin3 neck in the Head3/Neck1 chimera leads to a kinetic dead-end (intermediate 6′), where the motor transits into a weakly microtubule-bound state that is unable to produce processive hand-over-hand type motility and eventually detaches. The motor may still undergo few (3–4) ATPase cycles due to a transition from intermediate 6 back to 3. The coupling to motility is unclear.

In addition to information on the NcKin3 neck, our study also reveals important features of conventional kinesin's neck. In agreement with previous studies, the Head1/Neck3 construct (lacking the conventional neck domain) shows clear defects in processivity. Mutational analysis of the conventional kinesin neck suggested that the net charge [32], [33] as well as passive mechanical features (stiffness, in particular) affect the runlength of processive kinesins, and is optimized in the wildtype [22].

The influence of charges in the neck domain seems to be absent in the Head1/Neck3 chimera that moved processively, despite a net negative net charge of −3 (Figure 1). Also, specific interactions seem to be absent since none of the positive charges of uKHC suspected to mediate electrostatic contact is conserved. Mechanical features, however, seem to be important. The Head1/Neck3 chimera showed slower velocities and runlengths under all loads, indicating that the likelihood of forward stepping is generally diminished. According to our model, a force-dependent transition not connected to catalytic rates is specifically affected. Apparently, the wildtype neck of processive kinesins minimizes the diffusive search for the next microtubule-binding site. The coiled coil of NcKin3 may impair the positioning of the diffusing head in general, or specifically during phases of asymmetric hand-over-hand motility [38].

Our study identified the unit of motor core and neck-linker as crucial for normal stepping behavior. All constructs of this study contained motor core and neck-linker of the same parent motor, thus conserving potential interactions of neck-linker and motor core. Two important neck-linker residues (V_419_N_420_) are conserved between Kinesin-3 and Kinesin-1. These residues have been shown to stabilize the docked neck-linker conformation [39]. However, the residues preceding V_419_N_420_ are completely different, as well as the putative interaction partners on the motor core [23]. As the interaction of neck-linker and motor core seems to be crucial for controlling the transition from the double-head bound intermediate 6 to 7, the combination of motor core and neck-linker may be responsible for fully functional hand-over-hand motility. Alternatively, recent findings on conventional kinesin suggest that the kinesin-microtubule interface may respond to mechanical strain emerging in the “two-heads-bound” state [35]. Further studies on the mechanism of other ‘unconventional’ kinesins might shed light on this issue.

## Materials and Methods

### Cloning, Protein expression and purification

Wildtype reference constructs were prepared as described [40]. For the generation of the Head1/Neck3 chimera the N-terminal 342 amino acids of the NcKin436 proteins were amplified from the pT7-NKin436 expression vector by PCR. The reverse primer introduced a *Bsi*WI restrictions site at the C-terminus of the NcKin head domain that allowed replacement of the codons for the N-terminal 427 amino acids in pT7-NcKin3_558cys and pT7-NcKin3_558hTail plasmids.

For the generation of the Head3/Neck1 chimera the N-terminal 429 amino acids of NcKin3 were amplified by PCR on the basis of the pT7-NcKin3_558cys plasmid. Here, the reverse primer introduced a *Ngo*MIV restriction site at the C-terminus of the head domain that allowed replacement of the NcKin head domain in the pT7-NKin436 and pT7-NcKin436_hTail plasmids.

All constructs that do not contain the hTail sequence have a short peptide sequence added to the C-terminus that confers a reactive cysteine to the protein. This allows labeling with maleimide compounds (PSIVHRKCF, [41])

Expression and purification of the proteins was performed as described in [16]. Microtubules were prepared from pig brain tubulin [42], the Atto 488 and Biotin labeling of tubulin, and the polymerisation of microtubules were performed as described in [43]. For kinetic experiments microtubules were treated with apyrase 0.01 U/ml prior to centrifugation.

### Gliding assays

A flow cell was incubated for 5 min with hTail-tagged motors in motility buffer (10 mM MgCl_2_, 10 mM ATP, 100 mM KCl, 20 µM paclitaxel, 1 mg/ml BSA, 0.8 mg/ml casein in BRB80+ (80 mM PIPES·KOH, pH 6.8, 5 mM MgCl_2_, 1 mM EGTA)). After washing with blocking buffer (1 mg/ml BSA, 0.8 mg/ml casein in BRB80+), the flow chamber was filled with atto488-labelled microtubules in motility buffer. Both kinesin and microtubule solutions were supplied with an oxygen scavenging system (0.1 mg/ml glucose oxides, 0.02 mg/ml catalase, 2.25 mg/ml glucose). Gliding of the microtubules was observed total internal reflection microscope and their velocity was measured using the manufacturers' software (Olympus Biosystems GmbH, Planegg, Germany). For statistical analysis SigmaPlot 2000 Software (Systat, Point Richmont, CA, USA) was used.

### Single-molecule motility assays

Motors were labeled with the maleimide conjugate of the atto488 fluorophore (ATTO-tec GmbH, Siegen, Germany). Biotin-labeled microtubules were fixed on the surface of a flow chamber that was first incubated with 2 mg/ml BSA-biotin (Sigma-Aldrich Co., St. Louis, MO, USA) subsequently with 1 mg/ml Streptividin in BRB80+ buffer and 20 µM paclitaxel. After washing with 1 mg/ml BSA in BRB80+ motility mix (0.1–0.5 nM atto488-labeled kinesin, 20 µM–2 mM ATP, oxygen scavenger (see above), 0.2 mg/ml casein, 100 mM KCl in BRB80+) was flushed in. The gliding activity was observed in an Olympus IX71 TIRF microscope with an excitation wavelength of 488 nm and a Hamamatsu C-9100 front-illuminated CCD-camera. The optical resolution was 160 nm per 2×2-binned pixel, the integration time 200 ms.

### Steady-state ATPase

Microtubule activated steady-state ATP turnover rates were determined in a coupled enzymatic assay [44]. The assay was performed in 12A25+ buffer (12.5 mM Aces·KOH, 25 mM potassium acetate, 5 mM MgCl_2_, 0.5 M EGTA, pH 6.8) at 22°C. K_0.5,MT_ was determined at an ATP concentration of 1 mM. NcKin3 concentrations were typically 1 µM. To test whether the ATPase rates were affected by inactive motors in the protein preparations, we performed a microtubule binding and release step immediately before measuring microtubule-activated ATPase rates. ATPase rates measured on those preparations never deviated significantly from untreated preparations. This indicates that our preparations of wild-type and mutant kinesins contain mostly active protein.

### Pre-steady-state mant-ADP release

Kinesin constructs were incubated with equimolar amounts of mant-ATP (methyl-anthranoyl-ATP; Molecular Probes) for 30 min on ice. Pre-steady state mant-ADP release was measured in a SX-18MV Spectrometer (Applied Photophysics Ltd., Surrey, UK) where equal volumes (60 µl each) of 600 nM kinesin-mant-ADP are mixed with 0 to 55 µM microtubules and 2 mM ATP. Experiments were performed in 12A25+ buffer at 22°C. Fluorescence was excited at 356 nm and detected after passing a KV 450 filter (Schott AG, Mainz, Germany). The data were evaluated by double-exponential curve fitting using the TableCurve 2D software (Systat, Point Richmont, CA, USA).

The rates showed a hyperbolic dependence on the microtubule concentration and were fitted according to the equation:
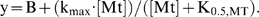
(1)


### Microtubule detachment

Microtubule detachment kinetics was determined via the change of light scattering in a stopped-flow assay. Kinesin-microtubule complex was formed by incubating Head3/Neck1 protein with a 1.5-fold excess of microtubules in the presence of 0.01 U/ml apyrase. Subsequently, 0.4 µM of the complex (final concentration) was mixed with ATP (0 to 1000 µM) in a stopped-flow apparatus (BioLogic Inc., Grenoble, France). The sample was illuminated at a wavelength of 436 nm and the light scattering signal observed through a 440±10 nm band-pass filter. At least five traces of each ATP concentration were used for averaging. Data were analyzed by single exponential curve fitting using the TableCurve2D software (Systat Software Inc., Point Richmond, CA, USA). The observed rates were plotted against the ATP concentration and fitted according to equation (2):
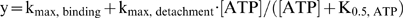
(2)


### Optical trapping

Optical trapping experiments were performed in a custom built optical trap described by Finer et al. [45]. Beads were captured in the beam of a 8 W Nd∶YAG laser (Coherent Deutschland GmbH, Germany) focused through a high numerical aperture objective (NA = 1.45, Olympus Deutschland GmbH, Germany). The position of trapped beads was detected by bright field imaging onto a quadrant diode (SPOT4D, UDT Sensors Inc., CA, USA). Samples were mounted on a piezo stage (P-517.3CL, Physik Instrumente GmbH, Germany) controlled by a feedback loop via a digital signal processor board (M62, Innovative Integrations, Ca, USA) enabling the detection of full runs of single motor molecules under constant force conditions. Data were acquired by an A/D converter board (NI-PCI-6259, National Instruments, Germany) with a sampling frequency of 40 kHz per channel and stored without prior filtering. The trap stiffness was calibrated for each trapped bead separately from the amplitude of the thermal diffusion and for some beads cross-checked by fitting a Lorentzian to the Power spectrum of the thermal diffusion [46]. Fluorescently labeled microtubules were visualized by total internal reflection microscopy with a high performance CCD camera (Pentamax Gen IV, Roper Scientific GmbH, Germany).

Fluorescently labeled microtubules were fixed to the glass surface of a flow chamber by the use of polyclonal tubulin antibodies (ab1289, Acris Antibodies GmbH, Germany) as described in [22] or by use of the biotin-streptavidin system (see above).

Kinesin molecules were allowed to adsorb to carboxylated polystyrene beads (532 nm, Polysciences Inc., USA) as described in [22].

To ensure data were acquired from single motor molecules we excluded all events from our analysis where more than 1/3 of the tested beads in a flow cell displayed movement when brought into contact with a microtubule [26].

Data from optical trapping experiments were analysed using IGOR Pro 4.01 (WaveMetrics, Portland, OR, USA). Runlengths were tabulated manually by analyzing intervals that started when the desired force was reached, and ended by dissociation of the bead from the microtubule. Velocities were calculated from 0.1 s phases of continuous movement. Stall forces were measured under saturating ATP levels without force-feedback. Events were considered a stall and included in the statistical analysis if the motor could hold a constant force for at least 50 ms before releasing from the microtubule. The stall force was measured by averaging over a 50 ms interval of the stall plateau and subtracting the average of a 50 ms interval of the base line.
